# Lithography Processable Ta_2_O_5_ Barrier-Layered Chitosan Electric Double Layer Synaptic Transistors

**DOI:** 10.3390/ijms22031344

**Published:** 2021-01-29

**Authors:** Sung-Hun Kim, Won-Ju Cho

**Affiliations:** Department of Electronic Materials Engineering, Kwangwoon University, 447-1, Wolgye-dong, Nowon-gu, Seoul 139-701, Korea; tjdgns0721@naver.com

**Keywords:** a-IGZO channel, chitosan electrolyte, high-*k* barrier layer, synaptic transistor, Ta_2_O_5_

## Abstract

We proposed a synaptic transistor gated using a Ta_2_O_5_ barrier-layered organic chitosan electric double layer (EDL) applicable to a micro-neural architecture system. In most of the previous studies, a single layer of chitosan electrolyte was unable to perform lithography processes due to poor mechanical/chemical resistance. To overcome this limitation, we laminated a high-*k* Ta_2_O_5_ thin film on chitosan electrolyte to ensure high mechanical/chemical stability to perform a lithographic process for micropattern formation. Artificial synaptic behaviors were realized by protonic mobile ion polarization in chitosan electrolytes. In addition, neuroplasticity modulation in the amorphous In–Ga–Zn-oxide (a-IGZO) channel was implemented by presynaptic stimulation. We also demonstrated synaptic weight changes through proton polarization, excitatory postsynaptic current modulations, and paired-pulse facilitation. According to the presynaptic stimulations, the magnitude of mobile proton polarization and the amount of weight change were quantified. Subsequently, the stable conductance modulation through repetitive potential and depression pulse was confirmed. Finally, we consider that proposed synaptic transistor is suitable for advanced micro-neural architecture because it overcomes the instability caused when using a single organic chitosan layer.

## 1. Introduction

Neural systems are efficient information-processing systems. Moreover, the mammalian brain contains approximately 100 billion neurons with 100 trillion synapses. With this particular network structure, our brain performs massively parallel and distributed computations by combining memory and processing capacities with an ultra-low power consumption of 20 W [1,2]. Inspired by this mechanism, the neuromorphic chip technology implemented in semiconductors is receiving increasing attention. In particular, synaptic transistors mimicking the synaptic behavior of the human brain are core elements of a neuromorphic chip [3]. The characteristics of these synaptic transistors can be emulated using an electric double layer (EDL) as the gate dielectric layer. High-density charges in the EDL accumulate even at very low voltages, which can respond to synaptic spikes through strong capacitance coupling effects [4,5]. A candidate for this EDL is the proton-conductive chitosan electrolyte with obvious advantages. Chitosan is obtained from chitin, the second most abundant organic biopolymer on the earth. As a biodegradable and renewable bio-friendly material, chitosan has great potential for skin-attachable and implantable neuromorphic chips [6]. Moreover, owing to the EDL effect of proton-conductive chitosan electrolyte, high gate capacitance (>1.0 µF/cm^2^) can be easily obtained from high-density mobile protons, enabling synaptic behavior [7,8]. Nevertheless, chitosan electrolytes suffer from processing capability inconveniences such as low chemical/mechanical resistance and ambient instabilities, which are limitations of organics materials. These limitations are rigorous obstacles in the implementation of practical electronic devices using chitosan. The application of organic chitosan EDL to transistors, which are basic components of electronic circuits, represents a major challenge in (complementary metal–oxide–semiconductor) CMOS process compatibilities.

Therefore, in this study, we propose amorphous In–Ga–Zn-oxide (a-IGZO) channel synaptic transistors gated by a high-*k* Ta_2_O_5_ barrier-layered organic chitosan EDL for a micro-neural architecture. The synaptic behaviors are emulated using the organic chitosan electrolyte EDL for polarization reaction by mobile protonic ions. Moreover, the inorganic high-*k* Ta_2_O_5_ barrier layer improves the chemical resistance and mechanical strength, enabling CMOS process compatibility and stably transferring presynaptic spikes to the postsynaptic a-IGZO channel. Ta_2_O_5_ is widely known as a biocompatible material and enhances its advantages in implementing human-friendly neuromorphic chips with chitosan [9,10]. Consequently, this study shows the possibility of implementing micro-neural structures and synaptic functions by overcoming the limitations of organic EDLs, which are expected to be useful for developing advanced neural networks. 

## 2. Results and Discussion

**Figure 1:**
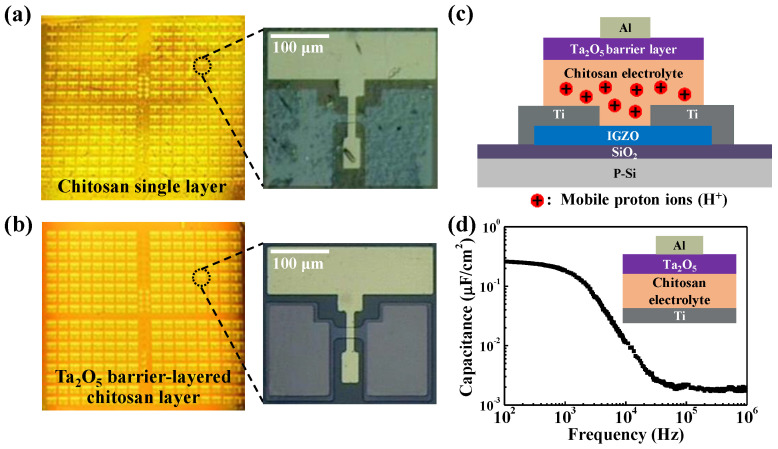
Optical microscope images of synaptic transistors fabricated by photolithography patterning process gated by (**a**) single chitosan layer and (**b**) high-*k* Ta_2_O_5_ barrier-layered chitosan layer; (**c**) cross-sectional schematics of Ta_2_O_5_–chitosan EDL transistor. (**d**) Frequency-dependent specific capacitance of the Ta_2_O_5_–chitosan electrolyte EDL capacitor.

Figure 1a shows the optical microscopic images of the top-gate structure chitosan electrolyte synaptic transistor without the Ta_2_O_5_ barrier layer. In the pattern image, the swelling and outgassing appearing in the chitosan EDL and source/drain (S/D) contact hole etching are incomplete due to the damaged chitosan EDL. In contrast, Figure 1b shows that the Ta_2_O_5_ barrier layer laminated chitosan electrolyte synaptic transistor can withstand the process of (ultraviolet) UV exposure and baking during the photolithography due to the Ta_2_O_5_ barrier. This implies that the Ta_2_O_5_ high-*k* thin film is an essential barrier ensuring the advanced patterning process by preventing damage to the organic chitosan electrolyte layer. Figure 1c shows the cross-sectional schematic diagrams of the top-gate structure Ta_2_O_5_ barrier-layered chitosan EDL synaptic transistor. Most of the previously reported chitosan-electrolyte synaptic transistors were fabricated by a simple patterning process using a shadow mask [11,12,13,14,15]. Figure 1d shows the specific capacitance of Ta_2_O_5_ barrier-layered chitosan electrolyte film as a function of frequency. It is observed that there is a strong electric-double layer (EDL) effect by mobile ion at the chitosan electrolyte interface with a high EDL capacitance of ~0.26 µF cm^−2^ at 100 Hz. Table 1 reports on the latest studies of synaptic transistors that applied chitosan electrolyte as an EDL. As the device is fabricated through a precise photolithography patterning process, it has a relatively small channel size compared to previous studies that applied a shadow mask.

**Figure 2:**
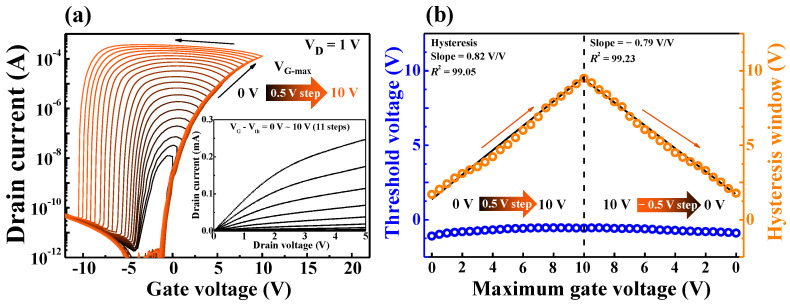
(**a**) Sequentially measured double-sweep I_D_−V_G_ curves, according to maximum gate voltage increases (0 to 10 V in 0.5 V increments); the inset shows the I_D_−V_D_ curves. (**b**) Threshold voltage and hysteresis window variation extracted from double sweep I_D_−V_G_ curves, according to maximum gate voltage increased and then decreased.

Figure 2a shows the double-sweep transfer characteristic (I_D_–V_G_) curves at a constant drain voltage (V_D_) of 1 V. The maximum gate bias (V_G___max_) increases positively (forward) from 0 to 10 V in increments of 0.5 V and sweeps back negatively (reverse). The counter-clockwise hysteresis in the double-bias I_D_–V_G_ curve increases with increasing V_G___max_ due to the slow polarization reaction by the mobile protons of the chitosan electrolyte [19]. The larger the V_G___max_, the larger the electric field, the stronger the dipole alignment, and the stronger the ion accumulation, resulting in a uniform increase in the hysteresis window. The inset in the figure shows the output characteristic (I_D_–V_D_) curves measured at V_G_–V_th_ from 0 to 10 V in increments of 1 V. As the V_D_ increases, the drain current increases linearly and then gradually saturates, indicating ohmic contact and pinch-off characteristics. Figure 2b shows the threshold voltage and hysteresis window of the double-sweep I_D_–V_G_ curve, according to V_G___max_. The threshold voltage remains almost constant as V_G___max_ varies, while the hysteresis window shows a slope of 0.82 V/V and linearity (*R*^2^) of 99.05 when V_G___max_ increases. Moreover, when V_G___max_ decreases, a slope of −0.79 V/V and an *R*^2^ of 99.23 can be obtained. These results suggest that the polarization response of the mobile protons is uniform in the Ta_2_O_5_ laminated chitosan EDL. 

**Figure 3:**
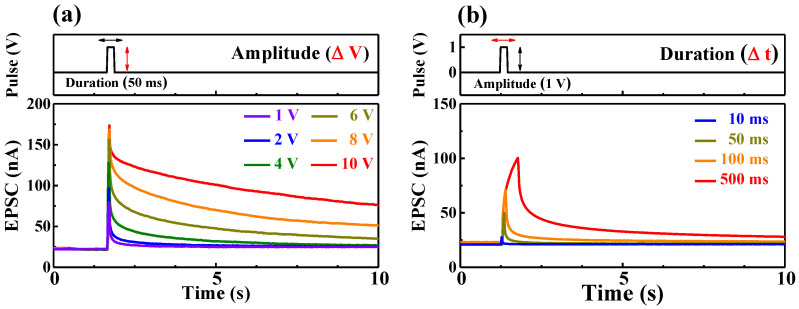
Excitatory postsynaptic current (EPSC) retention characteristics by a single gate pulse with (**a**) amplitude (1 to 10 V) variation for a fixed duration and (**b**) duration (10 to 500 ms) variation for a fixed amplitude.

In synaptic transistors, the gate voltage and channel conductance are considered as presynaptic stimulus and synaptic weight, respectively. The excitatory postsynaptic current (EPSC) caused by a single synaptic spike represents a fundamental neuromorphic property of the synaptic transistors. Moreover, a single synaptic spike repetition affects the postsynaptic short-term plasticity (STP) and the long-term plasticity (LTP) and significantly affects long-term weight formation [20]. Figure 3a,b shows the EPSC retention characteristics for a single gate pulse with amplitude (under fixed duration) and duration (under fixed amplitude) as variables, respectively. After a single gate stimulation, the EPSC of the postsynaptic channel gradually decreases from a peak value corresponding to the intensity of each stimulation. Furthermore, the magnitude of the residual EPSC increases as the amplitude and duration of the gate pulse increase. When the presynaptic spike amplitude and duration are low, the EPSC is maintained by the slow polarization reaction of the mobile protons inside the chitosan EDL. In contrast, when the spike amplitude is high, protons partially penetrate the a-IGZO channel layer. The increased channel conductance and long resting time due to electrochemical doping of the a-IGZO channel indicate the controllability of the synaptic weight from STP to LTP [21]. Therefore, the higher the pulse stimulation, the larger the weight capacity to mimic the behavior of the human brain. Therefore, the modulation of EPSC due to two or multiple consecutive spikes plays an essential function in decoding temporal information in biological systems [22]. 

**Figure 4:**
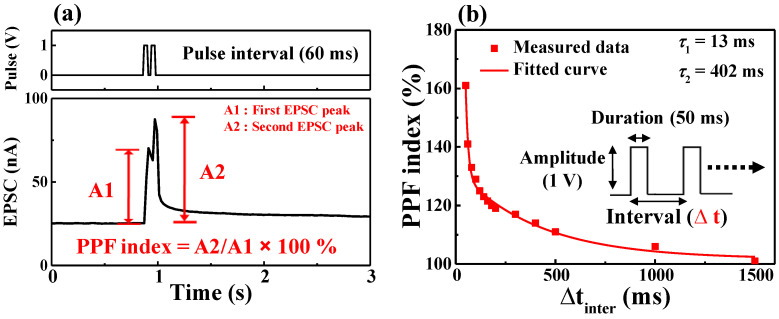
(**a**) EPSC triggered by a pair of pulses with a 60 ms interval; (**b**) paired-pulse facilitation (PPF) index (A2/A1) as a function of the pulse interval. The solid line corresponds to the fitting curve of the double-phase exponential function.

**Figure 5:**
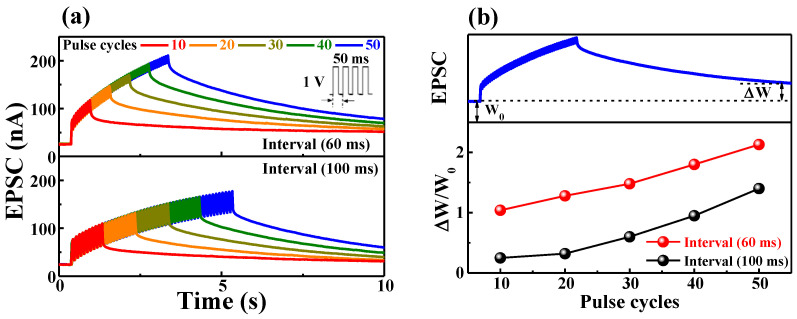
(**a**) EPSC recorded in response to presynaptic stimulation of multiple spikes (10–50 cycles); (**b**) change in synaptic weight according to the input pulse number. W0 is the initial EPSC before stimulation, and ∆W is the EPSC change after 10 s.

Figure 4a shows the EPSC triggered by two consecutive presynaptic spikes (1 V-amplitude, 50 ms-duration) with an interval of 60 ms. The paired-pulse facilitation (PPF) index corresponds to the amplification ratio between A1 and A2, the magnitude of the first and second EPSC peaks. Figure 4b shows the PPF index according to the interval time of two consecutive presynaptic spikes. At an interval of 50 ms, the PPF index is ~161%. However, above 1500 ms, the index decreases to ~100%. If the interval time is short, some mobile protons, accumulated at the interface between the electrolyte and the channel, do not have enough time to diffuse back before the arrival of the second spike. Therefore, the EPSC response of the second spike is stronger than that of the first spike [18]. The PPF index curve can be fitted considering the following double-phase exponential function [23,24]:

(1) $$PPF\;index=1+C_{1}\;exp(-\Delta t/\tau_{1})+C_{2}\;exp(-\Delta t/\tau_{2}),$$

where Δ*t* is the interval time between two consecutive presynaptic spikes. *C*_1_ and *C*_2_ are the initially facilitated magnitudes of the respective phases. *τ*_1_ and *τ*_2_ are the characteristic relaxation times, in this case, estimated as 13 ms and 402 ms, respectively. These results are similar to the time scale of biological synapses [25]. In a real synaptic operation, as the signals are transmitted and processed by multiple pulses [26], the identification of the postsynaptic response by multiple presynaptic pulses is essential. Figure 5a shows the EPSC response as a function of multiple presynaptic stimulation spikes at different pulse interval times. The EPSC value and resting time increase with the number of presynaptic spikes. In particular, multiple pulses with a short pulse interval of 60 ms have higher peak and residual EPSC values than those with a pulse longer than 100 ms. A practical method to identify a change in the synaptic response is by identifying the change in the synaptic weight. In artificial neural networks, the learning process is performed by adjusting synaptic weight values [27]. Figure 5b shows the change in the synaptic weight obtained by dividing the change occurring in the steady EPSC state after 10 s of the occurrence of stimulation (∆W) by that in the initial EPSC state before stimulation (W_0_) [28,29]. At the pulse interval of 100 ms, the change in the synaptic weight of 50 spikes is 1.4, while at the shorter interval of 60 ms, a significant change of 2.1 is observed. This result implies that a short pulse interval is insufficient for mobile protons to diffuse back before the next spike and successive pulses increase the synaptic weight. Thus, it is verified that the learning process of artificial neural networks could be controlled by adjusting the presynaptic spike interval.

**Figure 6:**
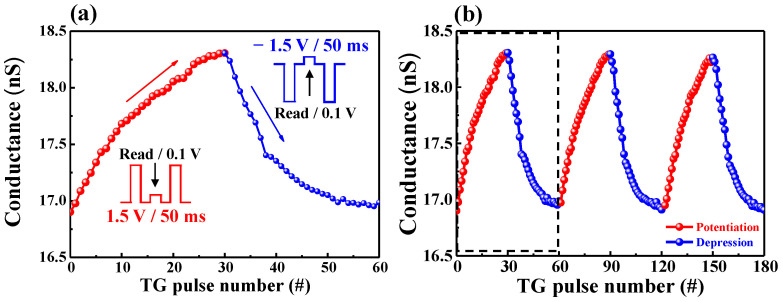
(**a**) Gradual conductance modulation by repeated synaptic potentiation (1.5 V for 50 ms) and depression (−1.5 V for 50 ms) pulses; (**b**) three cycles of repetitive conductance modulation.

Until now, we focused on the time-dependent learning mechanism. When this mechanism prologues, updating the weights is critical for matrix multiplication in artificial neural networks. They remain constant until the next update [30]. Figure 6a shows the continuous conductance modulation as a variation of the presynaptic pulse number. The steady conductance increment and decrement (indicating biological synaptic potentiation and depression) were evaluated by applying a repetitive positive presynaptic pulse (1.5 V for 50 ms), and negative presynaptic pulse (−1.5 V for 50 ms) at 0.1 V read voltage, respectively. Through 30 pulses, the conductance in the channel was modulated in the dynamic range of ~1.5 nS. Figure 6b shows the conductance retention when this potentiation and depression cycles are repeated three times. When comparing the first cycle and the third cycle, the conductance peak value was 99.7%, indicating stable operation.

## 3. Materials and Methods

### 3.1. Materials

A (100)-oriented p-type silicon wafer (resistivity range between 1−10 Ω·cm, LG SILTRON Inc., Gumi, Korea). IGZO sputter target (In_2_O_3_:Ga_2_O_3_:ZnO = 4:2:4.1 mol%, THIFINE, Incheon, Korea). 30:1 buffered oxide etchant (J.T. Baker, Phillipsburg, USA). Ti fillet (purity >99.999%, THIFINE, Seoul, Korea). Chitosan powder (from shrimp shell, deacetylation degree >75%, Sigma Aldrich Inc, Seoul, Korea). Acetic acid solution (purity >99%, Sigma Aldrich). Ta_2_O_5_ sputter target (THIFINE). Al fillet (purity >99.999%, THIFINE)

### 3.2. Fabrication Methods of Ta_2_O_5_ Barrier-Layered Chitosan EDL Transistor

A (100)-oriented p-type silicon wafer with a 100-nm-thick thermally grown SiO_2_ layer was cleaned following the standard (radio corporation of America) RCA process. For the channel layer (postsynapse), a 50 nm thick a-IGZO layer was deposited using radio frequency (RF) magnetron sputtering with an IGZO target (In_2_O_3_:Ga_2_O_3_:ZnO = 4:2:4.1 mol%). The active channel region with width/length = 20/10 μm for the a-IGZO synaptic transistor was developed by photolithography and wet etching with 30:1 buffered oxide etchant (BOE). For source/drain (S/D) electrodes, a 150 nm thick Ti film was deposited by an e-beam evaporator and patterned using the lift-off method. The organic chitosan EDL, the core material of the proposed synaptic device, was formed by the following procedure. The organic layer was formed using a 2 wt% chitosan electrolyte solution dissolved in 2 wt% acetic acids; the layer was spin coated, dried in air ambient for 24 h, and oven-baked at 130 °C for 10 min. The thickness of the chitosan EDL was 130 nm (±5 nm in deviation). A high-*k* Ta_2_O_5_ dielectric layer 80 nm thick was deposited by RF magnetron sputtering as a chemical/mechanical barrier layer of the organic chitosan electrolyte film. The top-gate electrode, serving as a presynapse, was formed using an e-beam evaporator and lift-off of a 150 nm thick Al film over the chitosan–Ta_2_O_5_ laminated gate dielectric. Finally, the S/D contact holes were opened by a reactive ion etching (RIE) process. Note that every patterning process for the fabrication of the organic chitosan–EDL-based synaptic transistors was performed by lithography.

### 3.3. Characterization of Devices

The optical microscope image of the fabricated Ta_2_O_5_ barrier-layered chitosan EDL transistor was analyzed using an SV−55 Microscope System (SOMETECH, Seoul, Korea). The frequency-dependent specific capacitance of Ta_2_O_5_–chitosan electrolyte EDL capacitor was analyzed using an 4284A Precision LCR meter (Hewlett-Packard Co., Palo Alto, CA, USA). The Transfer and output characteristics and synaptic behavior of Ta_2_O_5_ barrier-layered chitosan EDL transistor were measured using an Agilent 4156B Precision Semiconductor Parameter Analyzer (Hewlett-Packard Co., USA). The device measurement was conducted on a probe station in a dark box to avoid any light and electrical noises. To apply a presynaptic spike, electrical pulses were applied by Agilent 8110A Pulse Generator (Hewlett-Packard Co., USA).

## 4. Conclusions

In summary, we fabricated a top-gate structure synaptic transistor in which an inorganic Ta_2_O_5_ high-*k* thin film was laminated on an organic chitosan electrolyte EDL using a lithography process. We also showed artificial synaptic behavior based on protonic mobile ion polarization in chitosan electrolytes. The laminated Ta_2_O_5_ barrier layer effectively transmits top-gate (presynaptic) spikes to the chitosan electrolyte and a-IGZO channels (postsynaptic), while improving chemical resistance and mechanical strength for CMOS process compatibility. The double-sweep transfer curves of the a-IGZO synaptic transistor showed counter-clockwise hysteresis due to the uniform and stable polarization reaction and the back diffusion of mobile proton ions in the chitosan electrolyte. Moreover, the excitatory behavior control characteristics of the a-IGZO channel for artificial neural networks were verified by adjusting the presynaptic pulse amplitude, duration, and interval. Therefore, the proposed synaptic transistor is expected to be useful for implementing advanced micro-neural structure systems by providing CMOS process compatibility.

## Figures and Tables

**Table 1:** Benchmark of synaptic transistors using chitosan electrolyte as an electric double layer.

References	Gate Insulation Material	Structure Type	Channel Width/Length	Patterning Process
2020 Ref. [15]	Chitosan	Bottom-gate type	200 µm/200 µm	Shadow mask
2019 Ref. [16]	Chitosan	Bottom-gate type	1 mm/80 µm	Shadow mask
2018 Ref. [17]	Chitosan	Bottom-gate type	1 mm/80 µm	Shadow mask
2018 Ref. [18]	Chitosan	Bottom-gate type	1 mm/80 µm	Shadow mask
This study	Ta_2_O_5_ barrier Chitosan	Top-gate type	20 µm/10 µm	Photolithography

## Data Availability

The data presented in this study are available from the corresponding author upon reasonable request.
